# Spatially Explicit Environmental Factors Associated with Lymphatic Filariasis Infection in American Samoa

**DOI:** 10.3390/tropicalmed7100295

**Published:** 2022-10-12

**Authors:** Morgan E. Lemin, Angela Cadavid Restrepo, Helen J. Mayfield, Colleen L. Lau

**Affiliations:** School of Population Health, University of Queensland, Brisbane, QLD 4006, Australia

**Keywords:** lymphatic filariasis, environment, American Samoa

## Abstract

Under the Global Program to Eliminate Lymphatic Filariasis (LF) American Samoa conducted seven rounds of mass drug administration (MDA) between 2000 and 2006. Subsequently, the territory passed the WHO recommended school-based transmission assessment survey (TAS) in 2011/2012 (TAS-1) and 2015 (TAS-2) but failed in 2016, when both TAS-3 and a community survey found LF antigen prevalence above what it had been in previous surveys. This study aimed to identify potential environmental drivers of LF to refine future surveillance efforts to detect re-emergence and recurrence. Data on five LF infection markers: antigen, Wb123, Bm14 and Bm33 antibodies and microfilaraemia, were obtained from a population-wide serosurvey conducted in American Samoa in 2016. Spatially explicit data on environmental factors were derived from freely available sources. Separate multivariable Poisson regression models were developed for each infection marker to assess and quantify the associations between LF infection markers and environmental variables. Rangeland, tree cover and urban cover were consistently associated with a higher seroprevalence of LF-infection markers, but to varying magnitudes between landcover classes. High slope gradient, population density and crop cover had a negative association with the seroprevalence of LF infection markers. No association between rainfall and LF infection markers was detected, potentially due to the limited variation in rainfall across the island. This study demonstrated that seroprevalence of LF infection markers were more consistently associated with topographical environmental variables, such as gradient of the slope, rather than climatic variables, such as rainfall. These results provide the initial groundwork to support the detection of areas where LF transmission is more likely to occur, and inform LF elimination efforts through better understanding of the environmental drivers.

## 1. Introduction

Lymphatic filariasis (LF) is a neglected tropical disease that causes considerable disability and disfigurement in the mostly low-and-middle-income countries where it is endemic [1,2]. *Wuchereria bancrofti,* a filarial worm transmitted between humans by mosquitoes, is the parasite responsible for all LF cases in American Samoa [3]. Once the larvae enter the human host, they mature into adult filarial worms that damage the lymphatic system, potentially causing chronic pain and disability [4]. Chronic consequences of LF infection include lymphoedema, scrotal hydroceles and elephantiasis. There is also a major mental health burden stemming from social stigma and an inability to carry out employment and normal daily activities [5,6]. The World Health Organization (WHO) estimates that 863 million people worldwide remain at risk of LF infection, including the entire population of American Samoa [7].

The Global Program to Eliminate LF and the regional program, the Pacific Program to Eliminate LF (PacELF), were officially formed in 2000 and 1999, respectively [8,9]. These programs aim to eliminate LF as a public health problem using a two-pronged strategy. The first is to interrupt transmission using mass drug administration (MDA), followed by post-MDA surveillance using transmission assessment surveys (TAS). The second is the management of the chronic disease and disabilities already caused by LF [10]. From 2000 to 2022 these programs have delivered over 8.6 billion individual MDA treatments that have led to a 74% decline in infections globally [7]. Thanks to the success of elimination programs, eight countries in the Pacific region: Vanuatu, Niue, Cook Islands, the Marshall Islands, Tonga, Kiribati and Wallace and Fortuna, have had LF elimination status validated by the WHO. However, despite ongoing elimination efforts, transmission continues in some areas in the Pacific, including American Samoa, French Polynesia, and Fiji.

American Samoa had a baseline LF antigen prevalence of 16.5% in 1999, one of the highest in the region [11]. Following seven rounds of MDA between 2000 and 2006, school-based TAS in 2011/2012 (TAS-1) and 2015 (TAS-2) found LF antigen prevalence below the estimated threshold where ongoing transmission was considered unlikely (upper 95% confidence interval of <1% antigen-positive rate in areas where *Aedes* is the main vector) [10,12]. Following TAS-1 and TAS-2, subsequent surveys conducted outside PacELF activities found evidence of ongoing transmission [13,14] which was later confirmed by the 2016 ‘TAS Strengthening Survey’ that completed a community cluster survey alongside the third TAS (TAS-3) in American Samoa [15]. TAS-3, which sampled only six-and-seven-year-olds, found an adjusted antigen prevalence of 0.7% (95% CI 0.03–1.8). In the same time period, the community survey of those aged eight years and over found an even higher adjusted antigen prevalence of 6.5% (95% CI 4.5–8.6) [15]. 

In American Samoa, *Aedes polynesiensis* is the main LF vector, with *Ae. samoanus, Ae. upolensis* and *Ae. tutuilae* also responsible for LF transmission to a lesser extent. *Ae. polynesiensis* is a tropical mosquito species that does not survive in temperate regions or at high altitudes. They breed in still water inside small natural hollows, such as tree holes as well as in artificial containers such as discarded tyres [16]. Suitable environmental factors are integral to parasite development and mosquito breeding cycles. Mosquitoes will only hatch when submerged in water, and egg desiccation is generally fatal [17]. Conversely, intense rainfall causes breeding ground destruction and mechanical damage to the eggs [17]. Temperature impacts the timescale for parasite maturation, particularly in the extrinsic phase, and therefore parasite abundance [18]. *W. bancrofti* cannot survive at higher elevations, likely because the cooler temperatures do not support parasite maturation, while high slope gradients lead to increased water run-off, lessor pooling and mosquito breeding ground damage [1,19]. These environmental interactions are complex and dynamic, and relationships between environmental factors and LF infections have been found to vary between countries [20,21].

While TAS is the current standard surveillance tool, various alternative or complementary surveillance strategies are currently being explored to overcome the challenges of identifying residual LF infections in the post-MDA setting, when prevalence has reached very low levels. Potential methods include community surveys that include older age groups [15,22], molecular xenomonitoring (the detection of pathogen DNA in the mosquito) [23,24,25,26] and testing for alternative infection markers other than antigen, such as LF antibodies (Ab) [15,22,23,24,25,26,27,28,29]. The use of anti-filarial antibodies in post-MDA surveillance is currently being investigated in other studies [30]. The results are promising and suggest that Abs may provide earlier evidence of LF resurgence [30]. Another method to complement current surveillance strategies may lie in the understanding of the environmental drivers of transmission, and how this influences the geographic distribution of LF.

The geographical distribution of LF is determined by multifaceted interactions between mosquitos, parasites, and humans. Each of these interactions may be impacted by surrounding topographical, climatic, socio-demographic, and behavioural factors, leading researchers to believe that areas with a high risk of LF transmission could potentially be identified or predicted by understanding the environmental drivers [3,31,32]. This knowledge could help identify areas where transmission is more likely to persist (e.g., through spatial models), and where further targeted interventions may be required. To support and facilitate LF elimination in American Samoa, and to help develop more sensitive and targeted surveillance methods, the association between environmental factors and LF warrants further investigation. An understanding of these associations can also help reduce the risk of infections through management of environmental factors, such as landcover management. This study aims to identify potential environmental drivers of LF transmission in American Samoa by using spatial analysis to explore associations between LF seroprevalence in a 2016 community survey and locally collected or remotely sensed environmental data. While this study is focused on American Samoa, the methods could be widely applicable to other settings.

## 2. Materials and Methods

### 2.1. Study Location

**Figure 1:**
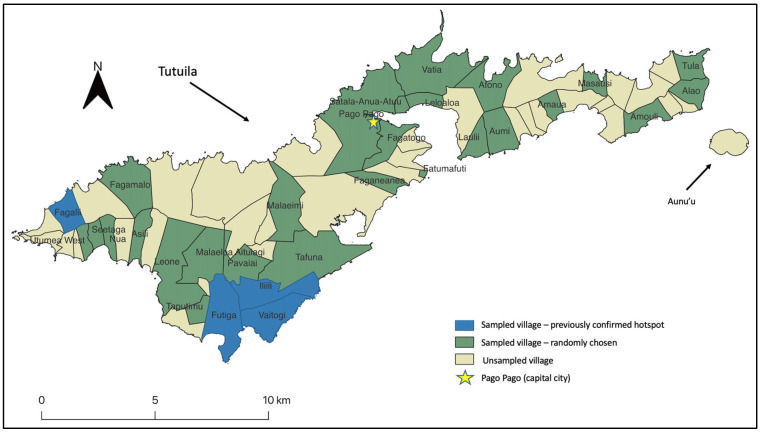
Map displaying administrative village borders on the two largest islands of American Samoa, Tutuila and Aunu’u. The locations of the villages sampled in the 2016 community survey are indicated on the map. Topographic base layer sourced from ESRI [37].

American Samoa is a US territory in the South Pacific that consists of five islands and two atolls [33]. It has a tropical climate with little variation in rainfall and temperature throughout the year [34]. The population is approximately 57,000, 95% of which live on the main island of Tutuila (shown in Figure 1) [35]. Tutuila is just over 30 km from east to west, with its highest peak at 653 m above sea level [36]. In total, the group of islands has an area of 197 km^2^ [35].

### 2.2. Data Collection

#### 2.2.1. Infection Markers

Data on LF infection markers were taken from the results of a 2016 community-based, population proportionate cluster survey conducted in American Samoa in parallel with TAS-3. The community household survey collected data on five serological markers as indicators of LF infection in participants aged eight years and over. These were microfilaremia (Mf) which indicates an active infection [38], circulating filarial antigen indicating the presence of living or dead worms [38], and three LF antibodies against Wb123, Bm14 and Bm33. Circulating filarial antigen and all three antibodies can persist to varying degrees both post-infection and post-treatment [30]. While the patterns and dynamics of antibodies have not been fully comprehended, it is understood that Wb123 appears in the early stages of infection while Bm14 and Bm33 antibodies may persist for multiple years following infection [38]. Circulating filarial antigen, and Wb123, Bm14 and Bm33 antibodies were measured using ELISA tests. The presence and density of Mf was determined through microscopy. The estimated prevalence of the five LF infection markers, along with laboratory methods, have previously been reported [13,15,38].

#### 2.2.2. Survey Design

The community survey included 30 primary sampling units (PSU). Each PSU included whole villages, combinations of small villages, or village segments with a total population of <2000 people. Two of these PSUs, Fagali’i and the contiguous villages of Futiga, Ili’ili, and Vaitogi, were included based on previous studies confirming high LF antigen prevalence [14]. The villages sampled in this study are displayed in dark green on the map of the two largest islands of American Samoa (Figure 1). Only the villages on the largest island of Tutuila were surveyed in 2016 and included in this study.

The sampling methodology of the 2016 community survey has been previously reported [15]. The end result of the sampling strategy was that 29% of the households in each PSU were randomly selected from a georeferenced list of buildings. All household members in the selected houses aged eight years and older were invited to participate. This selection process gave the total sample size (*n* = 2710), 98.5% of whom had their GPS coordinates registered based on their household location. The remaining 1.5% were assigned coordinates based on the village centroid. This ensured that every participant was assigned to the correct village. As these approximated locations were in the centre of the village, all were surrounded by other buildings, meaning that there was no effect on buffers used to calculate the environmental variables.

#### 2.2.3. Spatial Layers on Island, Village and Building Boundaries

Shapefiles of boundaries of islands, villages and buildings used in this analysis were compiled by the American Samoa Department of Commerce and downloaded from the American Samoa National Marine Sanctuary GIS data archive [39].

#### 2.2.4. Environmental Variables and Environmental Data

To select the environmental variables for analysis, we conducted a literature review on studies that investigated the relationship between LF infection markers and environmental factors. The review identified 27 studies from around the world. Information was collected on all the environmental factors reported in these studies, with particular focus on the strength and direction of each relationship (see Appendix A for details). The matrix also included study location and date, number of participants, study design, aims, main vector, and overall conclusions. The majority of the studies were conducted in Africa, with only a few undertaken in the Pacific region. As a consequence of this geographic imbalance, the main vector species studied was *Anopheles*, which has different breeding and biting habits to the *Aedes* mosquito in American Samoa. In the reviewed studies, there were an array of relationships reported between environmental variables and LF infection markers.

The basis for including environmental variables in this study were two-fold. First, environmental variables were selected based on the presence of strong evidence in the literature review indicating their relationship with the prevalence and distribution of various LF markers. Second, the environmental variable had to be suitable for analysis at a village scale. For example, it would not be logical to include the mean distance to a permanent waterbody aggregated to a village spatial level, as, upon visual inspection, most villages have waterbodies scattered throughout. Based on these criteria, 14 environmental variables were identified for inclusion in this study, however three had to be removed due to lack of available data with sufficient coverage. These three were annual average temperature, average temperature in the dry season and average temperature in the wet season.

Spatial environmental data for American Samoa were downloaded from multiple sources (Table 1). Datasets were selected to be as close to 2016 as possible to represent the environmental conditions at the time of the community survey. Datasets were excluded if there was insufficient coverage of American Samoa at a high enough resolution to represent the spatial heterogeneity within the small islands (≤1 km^2^). The issue of insufficient coverage applied mainly to temperature variables, which were removed as a covariate in this study.

#### 2.2.5. Environmental Data Collection and Extraction

**Figure 2:**
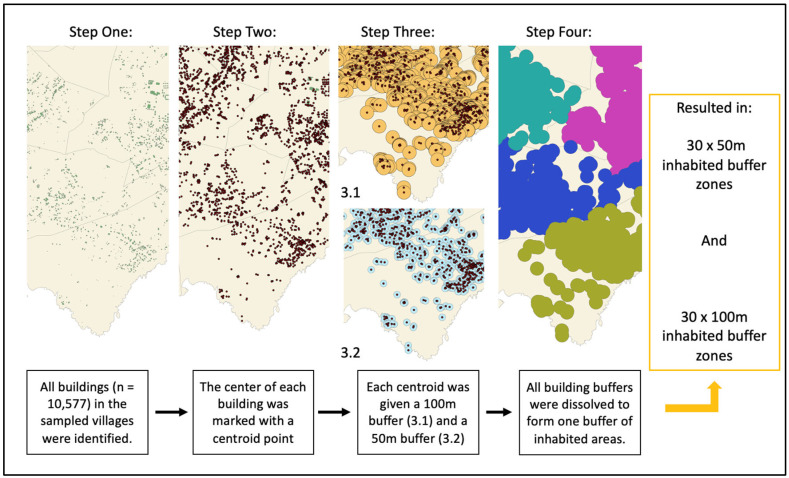
The process used to create the inhabited buffer zones using ArcGIS version 2.8.6 [47]. The building locations were acquired from the American Samoa National Marine Sanctuary GIS data archive [39]. Each colour in step four illustrates a different inhabited buffer zone.

To accurately represent the environmental factors in the inhabited areas of each village, where transmission is most likely, ‘inhabited buffer zones’ were created in ArcGIS Pro version 2.8.6 and used as the areas for environmental data extraction. By creating the inhabited buffer zones for each village, uninhabited areas, which are less likely to influence LF transmission, were removed from the analysis. To create the buffer zones, we assigned a centroid point to each building within the sampled villages using the Centroid (Polygon) tool in ArcGIS version 2.8.6 (Figure 2). Each centroid was then given a circular buffer, and all buffers in the village were dissolved using the ArcGIS version 2.8.6 dissolve tool to create a boundary of inhabited areas for the village. The buffer size for each building was first set at 100 m radius from the centroid, as 100 m is the approximate standard flight range of *Ae. Polynesiensis,* and is also a plausible representation of where people most frequently move around a building [46]. A second buffer size of 50 m radius was used for sensitivity analysis.

The spatial layers downloaded for elevation and population density did not need to be altered prior to extracting and averaging the environmental data per inhabited buffer zone. The remaining variables were derived and calculated from the downloaded layers using various methods, outlined below.

Normalised difference vegetation index (NDVI) is a measure of vegetation density and health, and was estimated from the Landsat Collection 2 Level-2: Surface Reflectance and Surface Temperature images [44]. All Landsat images from 2016 with a maximum cloud cover of <10% were included in the calculation. The raster calculator in ArcGIS version 2.8.6 was used to calculate the ratio of near-infrared bands, which are reflected by vegetation, and red light bands, which are absorbed by vegetation [47,48]. NDVI values range from −1 and +1. The values taken from ArcGIS version 2.8.6 were averaged over the year. A high NDVI in an inhabited buffer zone indicates high vegetation density, while a very low NDVI generally indicates water.

The downloaded landcover raster layer included ten landcover classes and needed to be reclassified to four separate rasters, one for each of the landcover classes that were chosen for inclusion in the analysis: rangeland, trees, crop land, and urban/built environments. These four classes were chosen based on the evidence found in the literature review that suggested they are most likely to have an association with LF infection markers. The remaining six landcover classes were not included in this analysis. The reclassified raster layer was converted into a polygon layer and the ArcGIS version 2.8.6 intersection tool was used to find the total area within each inhabited buffer zone that was covered by each of the landcover classes. The landcover class values used in the final models were the percentage of each inhabited buffer zone covered by that landcover class. The percentage of each landcover class was calculated by dividing the area of the buffer zone covered by the landcover class (m^2^) by the total area of the inhabited buffer zone. A description of each of the landcover classes used in this analysis is outlined in Table 2.

The slope gradient (degrees) was derived from the digital elevation model (DEM) raster layer using the ArcGIS version 2.8.6 slope analysis tool [47]. The monthly precipitation records for each month in 2016 were summed and averaged in ArcGIS version 2.8.6 to provide the annual rainfall in mm. Other variables calculated from this layer were the average monthly precipitation in the driest months (June to September) and wettest months of each year (October to May).

Spatial mean values of rainfall, elevation, slope gradient, NDVI and population density were calculated by inhabited buffer zone in ArcGIS software version 2.8.6 [47] to define the parameters for subsequent analyses.

### 2.3. Associations between Environmental Variables and LF Infection Markers

The statistical analysis of associations between the infection markers and environmental drivers was conducted using R studio software version 2022.02.2 [49]. Analysis was conducted at the village level, rather than household level, to best represent variation in the environmental factors. A descriptive statistical analysis was undertaken for each infection marker and environmental variable. The distribution of each variable was inspected using histograms, and the relationship between the five infection markers and the environmental variables was assessed using scatter plots. Village-level crude prevalence of Mf, LF antigen and each Ab (Wb123, Bm14, Bm33) were estimated, and binomial exact methods were applied to ascertain the 95% confidence intervals (95% CI). Choropleth maps were produced to visualise the estimated prevalence for each of the five infection markers in the sampled villages. For all remaining analysis and the final models, infection marker count data was used.

Each environmental variable initially had two sets of extracted data, using building buffer zones of 50 m and 100 m. The correlation between the environmental data extracted for the 50 m and 100 m buffers and all five infection markers was calculated and based on the Pearson correlation coefficient, the buffer size associated with a stronger correlation with each infection marker was used for the next step of the variable selection process.

After selecting one buffer size per environmental variable for each infection marker, all remaining independent variables were then examined for collinearity using a univariate Poisson regression model. Pairs with a correlation coefficient of >0.8 in the univariate Poisson models were considered highly correlated, and the variable with the highest Akaike information criterion (AIC) value in each pair was excluded from the final multivariable models.

As recent LF surveys in American Samoa suggest that each detection of infection marker is a discrete and relatively rare event, the final analysis for each marker used a Poisson regression model [14,15]. The final model selection and analysis for each infection marker was performed with a multivariable stepwise Poisson regression process that used the LF infection marker count data. The stepwise regression process using a forward selection of the predictor variables was used to find the most parsimonious model. A village population log offset was included in the model to account for differences in population size between sampled villages. The results of the multivariable Poisson regression models have been expressed as relative risk (RR), with the effect size calculated based on these values. For all analyses, a *p*-value of <0.05 was used to indicate statistical significance. A 95% confidence interval (95% CI) for RR that excluded 1 was also used to support the indication of significance.

## 3. Results

### 3.1. Village Level Prevalence of Infection Markers

The study included 2671 participants in 750 households spread across 30 villages. The infection marker with the highest prevalence in the sampled villages was the Bm33 Ab (45.63% 95% CI 43.73–47.45%), followed by Wb123 Ab (25.61% 95% CI 23.96–27.31%), Bm14 Ab (13.1% 95% CI 11.85–14.41%), antigen (5.05% 95% CI 4.25–5.95%) and Mf (1.27% 95% CI 0.88–1.8). The highest seroprevalence of LF was in the village of Fagali’i, a previously confirmed hotspot [14], where Bm33 Ab prevalence was 95.06% (95% CI 87.84–98.64%) and antigen prevalence 38.27% (95% CI 27.68–49.74%). Fagali’i also had the highest seroprevalence of Bm14 Ab and Wb123 Ab. Bm33 Ab was detected in all sampled villages. Conversely, Mf-positive persons were detected in only 11 of the sampled villages. A descriptive summary of the infection marker counts is shown in Table 3.

**Figure 3:**
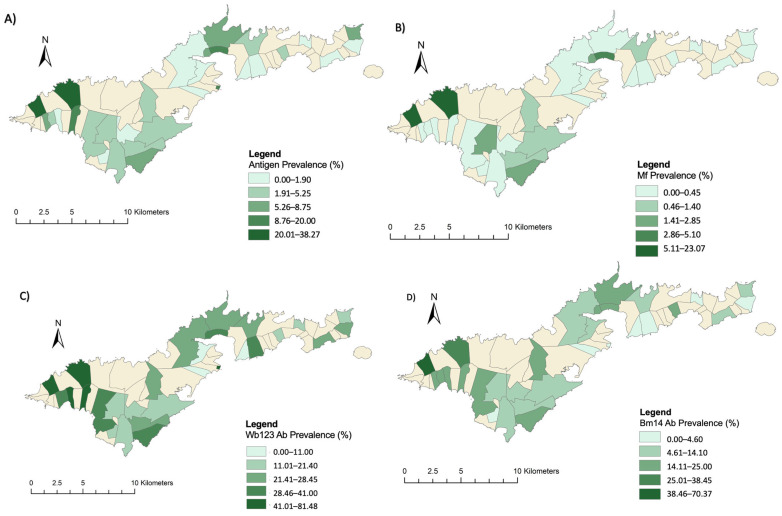

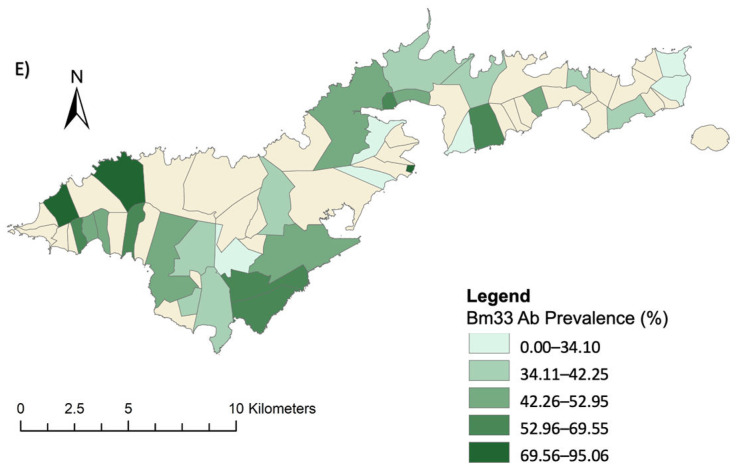
Crude prevalence (%) of each of the five infection markers sampled in the 2016 community survey in American Samoa: (**A**) antigen, (**B**) microfilaria, (**C**) Wb123 Ab, (**D**) Bm14 Ab, (**E**) Bm33 Ab.

The geographic distribution of LF seroprevalence amongst the sampled villages is shown in Figure 3. Areas of high antigen and Ab crude prevalence were consistently detected in the far western part of the island, particularly in the villages of Fagali’i, a previously confirmed hotspot, and Fagamalo [14]. The prevalence of Mf (confirmation of active infection) was also the highest in these two villages (14.81% and 23.08%). Conversely, the prevalence of all infection markers tended to be lowest in the eastern part of the island.

### 3.2. Village-Level Environmental Data

Annual rainfall, average monthly rainfall in the wet season and average monthly rainfall in the dry season were lowest in the inhabited buffer zone of Tula (in the east) and highest in the inhabited buffer zone in the combined area covering the villages of Satala-Anua-Atuu (central). Average annual rainfall in each inhabited buffer zone ranged between 2137 mm and 4526 mm. In most villages, the seasonal variation in monthly rainfall was minimal, with mean monthly rainfall varying by ~100 mm between the wet and dry season months. The population density ranged between 12 to 36 persons/km^2^ with an average of 18 persons/km^2^. As expected, both slope gradient and elevation were lower closer to the coastline and higher in the centre of the territory. There was minimal variation in the mean values of the environmental data extracted using 50 m and 100 m buffers (Table 3). The results of the environmental data extraction in each PSU, in both the 50 m and 100 m inhabited buffer zones, is reported in Appendix B.

### 3.3. Multivariable Poisson Regression Models

The models developed for each infection marker differed in the environmental variables that were found to be significant (Table 4). Tree cover, which was included in all five multivariable models, consistently had a positive association with infection marker positivity. The risk of Mf-positivity increased by 18% (95% CI 9%, 29% *p* < 0.01) for every 1% increase in tree cover in the inhabited buffer zone. Effect sizes of a similar magnitude were found in the other four infection marker models. The association between rangeland cover and Mf-positivity had the largest effect size of any relationship within the study. The risk of Mf-positivity was estimated to increase by 42% (95% CI 17%, 76% *p* < 0.01) for every 1% increase in rangeland cover. The Bm33 Ab model was the only Ab model to include rangeland cover, again finding a statistically significant positive relationship with Ab-positivity (5% 95% CI 2%, 8% *p* < 0.01). The percentage of crop cover within an inhabited buffer zone was included in the Wb123 Ab and Bm14 Ab models however the result in the Wb123 Ab model was not statistically significant. However, the Bm33 Ab model showed a statistically significant negative association between crop cover and Ab-positivity, whereby the risk of Ab-positivity decreased by 22% for every 1% increase in crop cover (95% CI −33%, −11% *p* < 0.01). Urban cover also had a statistically significant association with Mf-positivity and Ab-positivity.

Higher population densities had a negative association with infection marker positivity across all models, especially the Mf model (−12%, 95% CI −19%, −4% *p* < 0.01) and antigen model (−11% 95% CI −15%, −7% *p* < 0.01). There was also a negative association between steeper slope gradients and Mf (−9% 95% CI −16%, −2% *p* 0.02) and antigen (−9% 95% CI −12%, −6% *p* < 0.01) positivity.

In all models, rainfall showed no significant association with the detection of LF infection markers. NDVI had a very minimal association (RR between <0.0001 and 0.03) with infection marker positivity in all models where it was included (antigen, and Wb123, Bm14 and Bm33 antibodies).

## 4. Discussion

This study was the first to investigate associations between environmental variables and LF infection markers in American Samoa. We identified seven environmental variables that were associated with at least one of the five LF infection markers tested in the 2016 community survey in American Samoa. Each of the five infection markers were associated with a unique set of environmental variables, with the effect sizes varying between each marker. However, there were some overall consistencies detected in the results. In the villages included in this study, rangeland, tree cover, and urban cover had the strongest positive association with LF infection markers. Tree cover had a positive association with LF infection marker positivity in all five models. Similarly, higher population densities universally had a negative association with LF infection marker positivity. There is also evidence that slope gradient had a negative association with Mf and antigen positivity.

The associations between environmental variables and LF infection markers were generally consistent with the results found in previous studies. For example, the negative association with slope gradient in this study has been found almost universally in other studies conducted on the African continent [32,50]. However, while some associations were similar to reports from other countries, other findings were contradictory. The negative relationship between high population density and LF infection markers was opposite to the results found in most other studies.

Although the negative association observed with population density could appear to logically conflict with the positive association observed with urban landcover, it should be noted that the urban landcover class is not a direct measure of the level of urbanisation. More accurately, this landcover class represents evidence of the presence of manmade structures such as roads and buildings, which may have since been abandoned. In American Samoa, higher population density generally follows a similar distribution to the urban landcover classification but there are areas of medium-high population density that sit within rural areas. Additionally, large portions of the urban areas have low population densities. Areas with high population densities may have higher quality residential buildings and therefore less exposure to mosquitos. People in these areas might also have had better access during rounds of MDA. These possible confounding factors will require further investigation in the future.

The contrasting results between this study and studies in other locations may be related to the complexity of the dynamic relationships between LF transmission and environmental drivers in different locations. The differences observed between this study and other studies also demonstrate the importance of considering the specific geographical context for this type of analysis. Compared to other study locations, American Samoa is smaller in physical size and population and is characterised by minimal seasonal variability in climatic factors. Additionally, even the most densely populated areas of American Samoa would be considered as low density in most parts of the world.

Our study found that some landcover classes were strongly associated with the presence of LF infection markers. Rangeland produced the largest effect size in the study when analysed for its relationship with Mf-positivity, although the confidence interval was wide. Rangeland includes areas of shrubs, natural fields, and grassland [43]. This type of landcover may provide *Ae. polynesiensis* with the small puddles of water that it prefers to use as a breeding ground [16]. The tree class was another variable that had an association with infection marker positivity. Dense tree cover may protect mosquitos from fatally high temperatures and intense rainfall. This phenomenon was found in a similar study in Ghana, where mosquitos could survive higher temperatures if they were protected by dense tree coverage [50].

The crop class was included in two infection marker models and had a statistically significant negative relationship with Bm33 Ab-positivity within this study. Crop class was also included in the Wb123 Ab model but did not produce a statistically significant association (Table 4). A study in Burkina Faso found a similar negative relationship between crop cover and LF infections. They attributed it to the large quantities of insecticide use on the crops that temporarily reduces mosquito abundance [51]. However, their study was specifically related to cotton crops, and they suggested that different crop varieties may impact LF prevalence in different ways. Information on crop type was not available for this study.

Slope gradient showed a negative association with Mf-positivity and antigen-positivity, consistent with studies conducted in Nigeria and Ghana [32,50]. It is likely that steeper gradients promote stronger water run-off, which causes damage to mosquito eggs and the pools of water used for breeding grounds, leading to lower mosquito abundance [32]. It is also possible that slope gradient is acting as a proxy variable for elevation, as they were highly correlated in all models. A higher mean slope gradient is generally the result of high mean elevation. *W. bancrofti* cannot survive at high elevations, generally >600 m, as temperatures are outside of the species’ survival ranges [19,32,52]. In the villages sampled in this study, the highest mean elevation was in the inhabited buffer zone of Satala-Anua-Atuu (402 m). This makes it possible for temperature to be a confounding factor but potentially to a lesser extent than areas with mean elevations >600 m. In all models except the Bm14 model, elevation was not included due to multicollinearity with slope gradient.

This study did not include temperature as a variable due to the limited data, and rainfall was removed from the multivariable models due to multicollinearity, ill-fit or lack of statistical significance. Regardless, it is still possible that both rainfall and temperature play an important role in driving LF transmission. Lack of correlation does not always mean a lack of causation and there are strong, well-known biological pathways between temperature, rainfall and LF infection risk. These biologically plausible pathways indicate that further investigation is warranted in American Samoa when higher quality data become available. However, it is possible that the statistical correlation will not adequately represent the real-world dynamics due to the minimal variation in rainfall and temperature.

The main limitation of this study is the shortage of high-resolution datasets that completely cover American Samoa, which meant environmental variables with previously confirmed associations with LF in other parts of the world could not be analysed. The main example was temperature, where elevation had to be used as a proxy due to the limited coverage by the temperature raster layer. There was also no NDVI dataset which completely covers American Samoa. An NDVI layer was created to compensate for this absence, as described in Section 2. However, many LANDSAT images had to be excluded due to cloud coverage >10%, so the NDVI layer was not representative of the potential variation throughout the year. A further potential limitation is that migration and temporary residence were not considered. Previous investigations of travel impact on LF in in American Samoa have however found than recent travel was not related to LF infection [13,15]. 

This study included five markers as an indication of LF infection: antigen, Mf and three antibodies (Wb123, Bm14 and Bm33). Antigen remains the standard infection marker used in LF surveys but evidence is increasingly showing the value of including antibodies in surveillance [29]. However, there is still only a limited understanding of how antibody patterns change and develop over time [30]. As research into the dynamics of LF antibodies develops, it is possible that future surveys will test for antigens in combination with one or more antibodies [30]. Therefore, an understanding of how each individual infection marker is impacted by environmental factors is likely to prove useful. The challenge posed by interpreting the different positive counts in current TAS and community surveys is ongoing, and research is continuing.

It is outside the scope of this study to use the information on environmental associations to predict LF distribution in unsampled villages. However, it is encouraging to note the consistency between high LF prevalence and the presence of the potential environmental drivers when compared to the results of other studies around the world. In the two sampled villages with the highest LF prevalence, both had low average slope gradients, low population densities and high tree cover.

## 5. Conclusions

This study is the first step toward understanding the intricate associations between environmental factors and LF transmission in American Samoa. These results can be used in future spatial analysis of LF distribution, such as predictive risk mapping to identify potential hotspots and inform evidence-based strategies to strengthen surveillance. This type of analysis may also be used to assess the potential impact of climate change on LF transmission. Our models can be refined and adapted based on past, present and predicted future environmental data to understand changes in LF disease distribution as climate change continues to impact infectious disease distribution.

## Figures and Tables

**Table 1:** Datasets and sources for each environmental variable included in this study, with details of their spatial and temporal resolutions, American Samoa.

Variable Description	Dataset	Source	Spatial Resolution	Temporal Resolution
Temperature	MODIS/Terra Land Surface Temperature/Emissivity 8-Day L3 Global 1 km SIN Grid V06 [40]	USGS Earth Explorer	1 km	Weekly (2016)
Rainfall	Department of Commerce–Local Climate Data [41]	Pacific Environment Data Portal	1 km	Monthly (2016)
Elevation	NOAA–American Samoa 1/3 arc-second MHW Coastal Digital Elevation Model [42]	USGS Earth Explorer	10 m	1984-2012
Landcover	Sentinel-2 10 m Land Use/Land Cover Timeseries [43]	Esri	10 m	2017-2021
Surface reflectance (used to derive normalized difference vegetation index (NDVI))	Landsat Collection 2 Level-2 Surface Reflectance and Surface Temperature [44]	USGS Earth Explorer	30 m	Weekly (2016)
Population density	High Resolution Settlement Layer (HRSL) [45]	Pacific Data Hub based on Data for Good at Meta dataset using census data from 2010/2011	100 m	2020 (Based on 2011 census with a population growth rate of 0.23% applied)

**Table 2:** The four types of landcover analysed for their association with LF infection markers in American Samoa. Data were taken from the recently updated Esri Land Use/Land Cover 10 m map [43].

Landcover Class	Description
Crop	Areas with human planted vegetation below tree height.
Rangeland	Areas of shrubs, natural fields, and grassland, with no evidence of artificial plotting or tall tree coverage.
Trees	Areas of tall and dense vegetation, around 4.5 m or higher, including forests, swamps, mangroves, and savannas.
Built/Urban	Areas of human development including major paved roads, houses, towns, and large areas of asphalt.

**Table 3:** Descriptive statistics for rainfall (mm), elevation (m), slope gradient (degrees), normalised difference vegetation index (NDVI), landcover class (percent of inhabited buffer zone covered), population density (persons/km^2^) and counts of infection markers in a 2016 community survey, American Samoa.

Variable	Buffer Size	Mean	Standard Deviation	Minimum	Maximum
Village population	-	786.16	804.42	47.00	3195.00
Participants/village	-	89.03	81.10	5.00	307.00
Antigen (counts/village)	-	4.50	6.45	0.00	31.00
Mf (counts/village)	-	1.13	2.46	0.00	12.00
Wb123 Ab (counts/village)	-	22.80	21.32	2.00	83.00
Bm14 Ab (counts/village)	-	11.67	12.84	0.00	57.00
Bm33 Ab (counts/village)	-	40.63	40.17	4.00	175.00
Annual rainfall (mm)	50 m	3426.79	654.41	2136.74	4526.41
	100 m	3430.70	654.78	2128.86	4518.20
Average dry season rainfall per month (mm)	50 m	239.70	50.75	139.01	317.97
	100 m	240.10	50.95	138.28	317.21
Average wet season rainfall per month (mm)	50 m	351.70	63.60	230.54	461.31
	100 m	352.03	63.57	229.91	460.71
Average elevation (m)	50 m	95.51	95.66	0.00	402.41
	100 m	96.28	92.07	0.05	378.61
Average slope gradient (degrees)	50 m	15.96	11.54	0.00	39.17
	100 m	16.23	11.49	0.23	39.18
Average NVDI	50 m	0.34	0.05	0.21	0.41
	100 m	0.34	0.05	0.23	0.41
Crop cover in inhabited buffer zone (%)	50 m	0.11	0.34	0.00	1.78
	100 m	0.14	0.44	0.00	2.28
Tree cover in inhabited buffer zone (%)	50 m	26.35	18.89	0.29	66.59
	100 m	35.96	20.63	1.02	78.29
Rangeland cover in inhabited buffer zone (%)	50 m	5.36	5.79	0.00	21.09
	100 m	8.27	8.31	0.00	27.04
Urban cover in inhabited buffer zone (%)	50 m	59.10	21.88	16.48	95.54
	100 m	40.72	22.88	9.56	88.53
Average population density (person/km^2^)	50 m	20.30	6.74	12.00	36.00
	100 m	18.16	6.00	10.00	33.58

**Table 4:** Results of the multivariable Poisson regression models for the association between the five lymphatic filariasis infection markers sampled in the 2016 ‘TAS Strengthening Survey’ in American Samoa and environmental variables: rainfall (mm), population density (persons/km^2^), elevation (m), slope gradient (degrees), NDVI, landcover class (percent of village buffer zone covered), and LF infection markers.

	Microfilaria		Antigen		Wb123 Ab		Bm14 Ab		Bm33 Ab	
	RR (95% CI)	*p*-Value	RR (95% CI)	*p*-Value	RR (95% CI)	*p*-Value	RR (95% CI)	*p*-Value	RR (95% CI)	*p*-Value
Climatic Variables										
Annual Rainfall	-	-	-	-	-	-	1.0 (1.0, 1.0)	0.01	-	-
Dry Season Rainfall	-	-	-	-	-	-	-	-	-	-
Wet Season Rainfall	1.01 (1.0, 1.02)	<0.01	1.01 (1.0, 1.01)	<0.01	1.0 (0.99, 1.0)	0.1	-	-	1.0 (1, 1)	<0.01
Population Variables										
Population Density	0.88 (0.81, 0.96)	<0.01	0.89 (0.85, 0.93)	<0.01	0.96 (1.03, 1.06)	<0.01	0.96 (0.95, 0.98)	<0.01	0.98 (0.96, 0.99)	<0.01
Topographical and Landcover Variables										
Elevation	-	-	-	-	-	-	1.0 (1.0, 1.0)	0.04	-	-
Slope gradient	0.91 (0.84, 0.98)	0.02	0.91 (0.88, 0.94)	<0.01	1.01 (1.0, 1.02)	0.04	-	-	1.01 (0.99, 1.02)	0.09
NDVI	-	-	0.00 (0, 0.06)	<0.01	0.03 (0, 0.31)	<0.01	0.001 (0.0, 0.02)	<0.01	0.01 (0.01, 0.58)	<0.01
Crop class	-	-	-	-	0.85(0.71, 1.01)	0.07	-	-	0.78 (0.67, 0.89)	<0.01
Tree class	1.18 (1.09, 1.29)	<0.01	1.07 (1.04, 1.09)	<0.01	1.05 (1.03, 1.06)	<0.01	1.09 (1.07, 1.11)	<0.01	1.06 (1.05, 1.08)	<0.01
Urban class	1.09 (1.01, 1.18)	0.02	1.02 (0.99, 1.04)	0.17	1.02 (1.01, 1.04)	<0.01	1.04 (1.03, 1.06)	<0.01	1.04 (1.03, 1.08)	<0.01
Rangeland class	1.42 (1.17, 1.76)	<0.01	1.12 (1.06, 1.19)	0.03	-	-	-	-	1.05 (1.02, 1.08)	<0.01

RR = relative risk. Ab = antibody. CI = confidence interval. Values in bold indicate that the result was statistically significant based on effect level, 95% CI and a *p*-value of < 0.05. ‘-‘ indicates that the variable was not included in the model.

## Data Availability

The data used in the present study are available from the corresponding author on reasonable request.

## Appendix A. Summary of Studies Investigating Associations between Environmental Variables and Lymphatic Filariasis

**Table:**

Variable		*n* *	Study Reference	Location	Most Common Relationship Found
**Temperature**	**Day Land Surface Temperature**	4	Mwase et al., 2014 [53] Stensgaard et al., 2011 [2] Eneanya et al., 2018 [32] Kwarteng et al., 2021 [50]	Zambia Uganda Nigeria Ghana	Positive (*n* = 2)
	**Night Land Surface Temperature**	3	Mwase et al., 2014 [53] Stensgaard et al., 2011 [2] Kwarteng et al., 2021 [50]	Zambia Uganda Ghana	No relationship was found.
	**Mean max/min temperature**	2	Cano et al., 2014 [4] Eneanya et al., 2018 [32]	Global Nigeria	A positive relationship with mean minimum temperature.
	**Average Annual Temperature**	8	De Souza et al., 2010 [31] Manhenje et al., 2013 [1] Cano et al., 2014 [4] Stanton et al., 2013 [51] Eneanya et al., 2018 [32] Onapa et al., 2005 [54] Palayandi et al., 2014 [52] Slater & Michael 2013 [55]	Ghana Mozambique Global Burkina Faso Nigeria Uganda India African Continent	Positive, linear (*n* = 6)
**Land**	**Normalised Difference Vegetation Index (NDVI)**	8	Mwase et al., 2014 [53] Stensgaard et al., 2011 [2] De Souza et al., 2010 [31] Cano et al., 2014 [4] Stanton et al., 2013 [51] Eneanya et al., 2018 [32] Kwarteng et al., 2021 [50] Slater & Michael 2013 [55]	Zambia Uganda Ghana Global Burkina Faso Nigeria Ghana African Continent	Positive (*n* = 4) Did not contribute to the model (*n* = 3)
	**Soil pH**	1	Eneanya et al., 2018 [32]	Nigeria	No relationship found
	**Land Cover**	6	Mwase et al., 2014 [53] Cano et al., 2014 [4] Stanton et al., 2013 [51] Eneanya et al., 2018 [32] Kwarteng et al., 2021 [50] Rwegoshora et al., 2005 [56]	Zambia Global Burkina Faso Nigeria Ghana East Africa	Varied significantly depending on the type of landcover analysed.
**Water**	**Humidity**	2	De Souza et al., 2010 [31] Palayandi M. 2014 [52]	Ghana India	Positive (*n* = 1) Negative (*n* = 1)
	**Potential evapotranspiration**	1	Eneanya et al., 2018 [32]	Nigeria	Removed due to multicollinearity
	**Wetness Index**	2	Eneanya et al., 2018 [32] Grziwotz et al., 2018 (As dew point) [57]	Nigeria French Polynesia	Positive (*n* = 2)
	**Distance to waterbody**	8	Mwase et al., 2014 [53] Stensgaard et al., 2011 [2] Stanton et al., 2013 [51] Eneanya et al., 2018 [32] Nurjazuli & Santjaka 2020 [58] Chesnais et al., 2019 [59] Edirisinghe M. 2017 [3] Kwarteng et al., 2021 [50]	Zambia Uganda Burkina Faso Nigeria Indonesia Democratic Republic of the Congo Sri Lanka Ghana	Positive (*n* = 4) No relationship (*n* = 2)
	**Aridity**	2	Cano et al., 2014 [4] Eneanya et al., 2018 [32]	Global Nigeria	No relationship (*n* = 2)
	**Number of Months with Rainfall**	1	Stanton et al., 2013 [51]	Burkina Faso	Positive
	**Mean tidal level**	1	Grziwotz et al., 2018 [57]	French Polynesia	Positive
	**Rainfall/Precipitation**	13	Mwase et al., 2014 [53] Stensgaard et al., 2011 [2] De Souza et al., 2010 [31] Manhenje et al., 2013 [1] Cano et al., 2014 [4] Stanton et al., 2013 [51] Eneanya et al., 2018 [32] Kwarteng et al., 2021 [50] Hussaini et al., 2020 [60] Grziwotz et al., 2018 [57] Onapa et al., 2005 [54] Palayandi M. 2014 [52] Slater & Michael 2013 [55]	Zambia Uganda Ghana Mozambique Global Burkina Faso Nigeria Ghana Nigeria French Polynesia Uganda India African Continent	Positive, nonlinear (*n* = 7)
**Altitude**	**Elevation**	12	Mwase et al., 2014 [53] Stensgaard et al., 2011 [2] De Souza et al., 2010 [31] Manhenje et al., 2013 [1] Cano et al., 2014 [4] Stanton et al., 2013 [51] Eneanya et al., 2018 [32] Kwarteng et al., 2021 [50] Onapa et al., 2005 [54] Palayandi. M 2014 [52] Slater & Michael 2013 [55] Dhimal, Ahrens & Kuch 2014 [21]	Zambia Uganda Ghana Mozambique Global Burkina Faso Nigeria Ghana Uganda India African Continent Nepal	Negative, nonlinear (*n* = 8)
	**Slope**	2	Eneanya et al., 2018 [32] Kwarteng et al., 2021 [50]	Nigeria Ghana	Negative (*n* = 2)
**Human Factors**	**House type**	1	Srividya et al., 2018 [61]	India	The proportion of concrete and tiled, not thatched, houses was higher in hotspots (31.8% and 47.3%)
	**Water in the house**	2	Nurjazuli & Santjaka 2020 [58] Hussaini et al., 2020 [60]	Indonesia Nigeria	Positive (*n* = 2)
	**Distance to stable light**	2	Eneanya et al., 2018 [32] Kwarteng et al., 2021 [50]	Nigeria Ghana	Negative (*n* = 2) LF prevalence decreased with increasing distance

* The total number of studies included in the literature review that analysed the variable.

## Appendix B. Extracted Environmental Data for Normalised Difference Vegetation Index (NDVI), Rainfall (mm), Population Density (persons/km^2^), Elevation (m), Slope Gradient (degrees), and Landcover Class (Percent of Inhabited Buffer Zone covered) in Each Village Inhabited Buffer zone of the 2016 Lymphatic Filariasis Community Survey, American Samoa

**Table:**

	Average Normalised Difference Vegetation Index		Annual Rainfall (mm)		Average Rainfall in Dry Months (mm)		Average Rainfall in Wet Months (mm)		Average Population Density (Persons/km^2^)		Average Elevation (m)		Average Slope Gradient (Degrees)		Crop Cover in Inhabited Buffer Zone (%)		Forest Cover in Inhabited Buffer Zone (%)		Rangeland Cover in Inhabited Buffer Zone (%)		Urban Cover in Inhabited Buffer Zone (%)	
**VILLAGE**	50 m	100 m	50 m	100 m	50 m	100 m	50 m	100 m	50 m	100 m	50 m	100 m	50 m	100 m	50 m	100 m	50 m	100 m	50 m	100 m	50 m	100 m
**Afono**	0.4	0.4	3447.1	3422.3	245.5	243.0	352.9	350.7	22.6	19.1	18.1	20.3	10.1	10.0	0.0	0.0	51.8	61.8	1.4	3.2	16.5	9.6
**Alao**	0.4	0.4	2280.8	2281.6	152.0	152.0	239.4	239.4	26.5	23.9	63.5	58.8	16.1	16.0	0.0	0.0	28.5	35.9	5.5	7.5	62.7	43.3
**Amaua**	0.4	0.4	3291.9	3298.6	230.4	231.1	338.9	339.5	17.8	16.0	159.2	168.5	25.3	27.2	0.0	0.0	35.0	40.3	7.2	18.8	52.0	26.7
**Amouli**	0.3	0.3	2467.6	2461.5	166.0	165.6	258.5	257.8	18.9	17.9	94.2	92.9	22.5	22.4	0.0	0.0	38.5	49.2	9.9	11.7	45.0	24.6
**Asili**	0.3	0.3	3877.8	3941.9	287.6	293.2	390.5	396.5	16.0	15.3	89.5	101.7	23.4	24.4	0.0	0.0	46.3	65.0	0.0	0.0	43.6	21.1
**Aumi**	0.3	0.3	3755.6	3740.4	265.9	265.0	384.2	382.6	21.5	18.9	132.4	137.0	29.0	28.5	0.0	0.0	15.1	26.4	20.3	27.0	56.2	28.4
**Fagalii**	0.4	0.4	2828.0	2842.5	195.3	196.5	292.6	293.9	12.0	10.8	8.7	9.2	4.5	5.7	0.0	0.0	66.6	78.3	0.0	0.0	31.8	17.7
**Fagamalo**	0.4	0.4	2985.0	2990.1	206.0	206.5	308.4	308.9	12.7	12.7	0.1	0.9	0.5	1.9	0.0	0.0	60.6	69.1	0.0	0.0	35.6	18.8
**Faganeanea**	0.3	0.3	4173.3	4155.6	290.9	289.2	427.0	425.3	21.7	17.0	112.0	115.0	31.7	31.7	0.0	0.0	42.8	42.4	10.2	16.3	32.4	15.2
**Fagatogo**	0.3	0.3	4393.5	4369.5	304.3	302.6	447.8	445.6	36.0	33.6	2.3	4.6	2.4	3.7	0.0	0.0	8.9	14.5	5.8	8.0	77.1	60.3
**Fatumafuti**	0.3	0.3	3224.8	3230.2	210.5	211.0	335.5	336.0	30.0	30.0	0.0	0.0	0.0	0.2	0.0	0.0	23.3	27.9	8.1	13.1	59.6	28.1
**Futiga**	0.4	0.4	3281.0	3266.0	229.0	227.7	335.3	334.0	16.6	13.6	93.3	92.1	14.6	13.1	0.0	0.1	28.4	45.2	3.2	4.7	67.5	48.9
**Iliili**	0.4	0.4	3121.1	3113.8	212.9	212.3	324.0	323.4	21.5	18.8	60.0	57.4	3.0	2.9	0.0	0.1	3.3	6.4	3.8	9.3	92.1	82.4
**Laulii**	0.3	0.3	3563.8	3536.3	245.9	243.3	367.0	364.4	26.7	22.3	95.3	93.9	22.7	23.1	0.1	0.0	10.5	14.2	12.2	22.7	70.2	44.6
**Leloaloa**	0.3	0.2	4390.9	4394.9	307.4	307.8	452.3	452.5	18.4	16.0	374.8	361.3	35.0	37.2	0.5	0.3	11.6	17.1	21.1	25.0	22.9	14.6
**Leone**	0.3	0.3	3219.0	3315.4	226.7	235.5	329.2	338.0	17.4	16.2	42.7	54.9	8.2	9.2	0.0	0.0	10.3	19.5	1.4	2.0	85.9	73.8
**Malaeimi**	0.4	0.4	4188.3	4212.7	299.7	301.9	423.0	425.3	14.8	13.4	97.1	98.2	18.2	18.2	1.8	2.3	14.0	25.8	2.6	5.2	78.2	62.4
**Malaeloa Aitulagi**	0.4	0.4	3849.7	3932.0	281.5	289.0	388.0	395.6	23.2	19.1	106.7	133.6	13.9	16.4	0.0	0.0	34.9	52.7	3.5	5.6	59.2	39.3
**Masausi**	0.3	0.4	2425.9	2438.0	165.3	166.1	252.3	253.5	16.7	17.5	13.1	16.1	6.1	7.2	0.0	0.0	57.1	71.8	4.7	3.4	33.9	13.6
**Nua**	0.3	0.3	3734.3	3733.2	275.3	275.3	377.0	376.8	12.6	11.6	116.1	115.9	27.4	26.2	0.0	0.0	17.2	32.6	3.8	2.7	59.8	35.9
**Pago Pago**	0.3	0.4	4269.6	4274.0	297.6	298.0	436.3	436.7	31.8	29.0	164.1	164.2	26.4	25.8	0.0	0.0	14.1	28.3	1.8	5.6	80.9	59.6
**Pavaiai**	0.4	0.4	4042.2	4048.7	293.2	294.1	407.7	408.3	12.4	11.5	130.4	133.8	8.1	8.5	0.1	0.2	10.3	19.1	0.5	1.6	75.1	63.3
**Satala-Anua-Atuu**	0.2	0.2	4526.4	4518.2	318.0	317.2	461.3	460.7	30.4	27.0	402.4	378.6	38.8	39.2	0.0	0.0	1.7	6.0	16.2	26.3	65.4	44.4
**Seetaga**	0.3	0.3	3818.9	3772.2	283.3	279.5	384.4	379.9	12.5	11.6	163.2	165.8	29.0	29.4	0.0	0.0	25.8	38.9	0.1	0.1	54.0	30.0
**Tafuna**	0.3	0.3	3125.5	3122.3	210.9	210.8	327.2	326.7	23.1	21.5	28.1	27.7	3.4	3.4	0.5	1.0	0.3	1.0	2.8	7.2	95.5	88.5
**Taputimu**	0.4	0.4	2983.3	2995.9	203.0	204.1	307.7	308.9	14.9	14.1	35.3	35.9	1.8	1.7	0.1	0.1	12.2	22.7	0.2	1.6	87.3	75.5
**Tula**	0.4	0.4	2136.7	2128.9	139.0	138.3	230.5	229.9	30.3	23.5	18.0	16.7	9.6	8.9	0.0	0.0	19.5	32.7	6.1	10.3	70.6	42.7
**Utumea West**	0.4	0.3	3371.0	3376.7	246.3	246.8	341.7	342.2	12.0	10.0	160.3	155.6	24.7	24.3	0.0	0.0	57.6	58.7	0.0	0.0	23.2	10.1
**Vaitogi**	0.4	0.4	2838.4	2842.5	188.4	188.8	298.5	298.8	15.3	14.0	38.2	40.4	2.3	2.6	0.2	0.2	14.8	27.1	1.3	2.1	82.7	66.8
**Vatia**	0.4	0.3	3192.1	3165.1	213.3	211.6	331.9	329.1	23.1	19.0	46.0	37.3	20.0	17.7	0.2	0.1	29.4	45.0	7.5	7.0	56.1	31.5
